# Effect of *Lactiplantibacillus plantarum* cell-free culture on bacterial pathogens isolated from cystic fibrosis patients: *in vitro* and *in vivo* studies

**DOI:** 10.3389/fmicb.2024.1440090

**Published:** 2024-09-16

**Authors:** Carla Luciana Abán, Silvia Orosco, Julio Nicolás Argañaraz Aybar, Leonardo Albarracín, Analía Venecia, Liliana Perret, Sonia Ortiz Mayor, Keita Nishiyama, Juan Carlos Valdéz, Haruki Kitazawa, Julio Villena, Nadia Gobbato

**Affiliations:** ^1^National Council of Scientific and Technological Research (CONICET)–CCT (Salta-Jujuy), Salta, Argentina; ^2^Pneumonology Department, Niño Jesus Children Hospital, SIPROSA, Tucuman, Argentina; ^3^Laboratory of Immunology, Faculty of Biochemistry, Chemistry and Pharmacy, National University of Tucuman, Tucuman, Argentina; ^4^Laboratory of Immunobiotechnology, Reference Centre for Lactobacilli (CERELA-CONICET), Tucuman, Argentina; ^5^Institute of Maternity and Gynecology “Nuestra Señora de las Mercedes”, SIPROSA, Tucuman, Argentina; ^6^Rehabilitation Department of the Integrated Health Program of the Ministry of Health of the Tucuman Province, Tucuman, Argentina; ^7^Hospital Centro de Salud “Zenon Santillan”, SIPROSA, Tucuman, Argentina; ^8^Food and Feed Immunology Group, Laboratory of Animal Food Function, Graduate School of Agricultural Science, Tohoku University, Sendai, Japan; ^9^Livestock Immunology Unit, International Education and Research Center for Food and Agricultural Immunology (CFAI), Graduate School of Agricultural Science, Tohoku University, Sendai, Japan

**Keywords:** *Lactiplantibacillus plantarum*, supernatant, respiratory infection, *Pseudomonas aeruginosa*, cystic fibrosis

## Abstract

This study aimed to investigate the effects of the cell-free supernatant of *Lactiplantibacillus plantarum* ATCC^®^ 10241^TM^ on the biofilm-forming capacity of *Pseudomonas aeruginosa* strains isolated from cystic fibrosis (CF) patients. In addition, the study evaluated the *in vivo* potential of the cell-free supernatant to modulate inflammation and reduce lung damage in mice infected with *P. aeruginosa* strains or co-challenged with *P. aeruginosa* and the *Streptococcus milleri* group (SMG). The results showed that CF-derived *P. aeruginosa* strains can infect the respiratory tract of adult mice, inducing local inflammation and lung damage. The severity of these infections was exacerbated when *P. aeruginosa* was co-administered with SMG. Notably, nebulization with the cell-free supernatant of *L. plantarum* produced beneficial effects, reducing respiratory infection severity and inflammatory responses induced by *P. aeruginosa*, both alone or in combination with SMG. Reduced bacterial loads and lung damage were observed in supernatant-treated mice compared to controls. Although further mechanistic studies are necessary, the results show that the cell-free supernatant of *L. plantarum* ATCC^®^ 10241^TM^ is an interesting adjuvant alternative to treat *P. aeruginosa* respiratory infections and superinfections in CF patients.

## Introduction

*Pseudomonas aeruginosa* is an opportunistic pathogen that can cause chronic airway infections in patients with cystic fibrosis (CF; Sala and Jain, 2021). The infections produced by *P. aeruginosa* in CF patients have been marked with the highest priority for surveillance and epidemiological research (Horcajada et al., 2019; Reynolds and Kollef, 2021; Souza et al., 2021), considering that this bacterium is a global threat because of its ability to become increasingly resistant to all available antibiotics (Tümmler, 2019; Laborda et al., 2022). *P. aeruginosa* infection can occur as early as the 1st year of life, is usually non-mucoid, and evolves over time, accumulating a series of adaptations to the CF lung. These adaptations include a mucoid phenotype, antibiotic resistance, and a loss of acute virulence factors, among others, and contribute to airway persistence and chronic pathogenicity (Gibson et al., 2003). The biofilm mode of life is considered important for the persistence of *P. aeruginosa* during long-term colonization of CF airways (Rossi et al., 2020). Biofilm synthesis and virulence factors produced by *P. aeruginosa* are regulated by chemical signals, including acyl-homoserine lactones (AHL) secreted by bacteria. When bacteria reach a threshold number, a concentration of signal molecules is reached, triggering the expression of the genes involved in synthesizing biofilm and virulence factors (Reading and Sperandio, 2006). Then, biofilm formation is a key event for establishing a chronic infection in the CF lung, promoting an accelerated decline of pulmonary function.

It is considered that respiratory infections in CF patients are usually polymicrobial. Historically, streptococci isolated from respiratory samples of CF patients were considered contaminants from the sampling process and were not recognized as clinically relevant. However, these bacteria are increasingly recognized as members of the CF lung microbiome and are associated with enhanced inflammation and lung damage (Sibley et al., 2008; Scott and O'Toole, 2019). Notably, the presence of the *Streptococcus milleri* group (SMG; also known as the anginosus group streptococci) can enhance the exacerbation and lung damage in CF patients infected with *P. aeruginosa* (Duan et al., 2003; Scott and O'Toole, 2019). It was reported that streptococci influence the growth and virulence of *P. aeruginosa*, potentiating its ability to damage lung tissue (Sibley et al., 2008; Scott and O'Toole, 2019). SGM can produce enzymes that can damage tissue and form abscesses, such as hyaluronidase, DNase, RNase, gelatinase, and collagenase, that may promote infections with other bacteria such as *P. aeruginosa* (Sibley et al., 2008). Given the well-documented nature of polymicrobial infections in the context of CF, strategies to prevent or treat infections with *P. aeruginosa* should also consider other microorganisms, such as SMG.

An attractive therapy to prevent *P. aeruginosa* chronic infection is bacteriotherapy, which uses beneficial harmless bacteria from the commensal microbiota, including lactobacilli. Several studies have reported the ability of lactobacilli strains to reduce *P. aeruginosa* growth, biofilm formation, and virulence. It was shown that culture supernatants of two human probiotic bacteria, *Lacticaseibacillus casei* CRL431 and *Lactobacillus acidophilus* CRL730, can attenuate *P. aeruginosa* biofilm and virulence by inhibition of the quorum sensing (Díaz et al., 2020). Similar effects were described for other lactobacilli strains (Shokri et al., 2018; Azami et al., 2022). Notably, most of the studies evaluating the effect of lactobacilli were performed with *P. aeruginosa* strains isolated from chronic wounds, while the interaction of lactobacilli and pathogenic bacteria isolated from CF patients was less explored. Recent studies demonstrated that some lactobacilli could inhibit the planktonic growth of *P. aeruginosa*, while others can reduce the pre-formed biofilm in an artificial sputum medium that mimics the CF lung microenvironment (Batoni et al., 2023; Pompilio et al., 2024). These previous studies indicate that lactobacilli and their metabolites are promising candidates as adjuvants in the antimicrobial therapy of *P. aeruginosa* infections in CF patients.

In a previous study, we demonstrated that the culture supernatant and the acid filtrate of *Lactiplantibacillus plantarum* ATCC^®^ 10241^TM^ grown in De Man–Rogosa–Sharpe (MRS) medium can inhibit the multiplication and viability of *P. aeruginosa in vitro*, reducing the synthesis of AHL, virulence factors, and the production of biofilm (Valdéz et al., 2005). Furthermore, the *in vivo* application of *L. plantarum* to burns infected with *P. aeruginosa* in a murine model reduced its colonization, regulating the inflammatory response and promoting tissue repair (Valdéz et al., 2005). When the culture supernatant of lactobacilli was analyzed, we found several compounds with biological activity, such as organic acids with low molecular weight (lactic, butyric, acetic, and succinic acids), H_2_O_2_, alcohols, benzoic acid, 5-methyl hydantoin, 2,5-mevalonolactone, and isobutyl piperazinedione. Many of them have proved to have growth inhibitory activity on Gram-negative bacteria and to synergize with each other in their antimicrobial action (Ramos et al., 2015). Notably, the presence of AI-2, which is considered an inhibitor of AHL, was determined in the *L. plantarum* supernatant by GC-MS, and its biological activity was confirmed by the *Vibrio Harveyi* bioassay (Ramos et al., 2015). Based on these observations, we used the supernatants of *L. plantarum* as clinical treatment by topical applications on infected chronic wounds, including diabetic foot ulcers, burns, and venous ulcers (Peral et al., 2009, 2010; Aybar et al., 2022). This treatment was able to reduce or eliminate *P. aeruginosa*, as well as diminish the amount of necrotic tissue in the wound area. Interestingly, the treatment promoted wound healing with increased production of TGF-β, IL-8, and IL-8-R (Peral et al., 2009, 2010), indicating the capacity of the culture supernatant to modulate the local immune response.

This study aimed to investigate the effect of the cell-free supernatant of *L. plantarum* ATCC^®^ 10241^TM^ on the capacity of *P. aeruginosa* strains isolated from CF patients to form biofilms. In addition, the study evaluated *in vivo* the ability of the cell-free supernatant to modulate respiratory inflammation and reduce lung damage in mice infected with the *P. aeruginosa* strains or challenged with *P. aeruginosa* and SMG.

## Materials and methods

### Bacterial strains

Bacterial strains were isolated from sputum samples of CF patients using standard clinical microbiological methods (Delgado et al., 2018). *P. aeruginosa* and SMG (*S. anginosus*) isolated from CF patients were used in this study. Analysis of the 16s RNA nucleotide sequence of the isolated strains showed that they were not genetically related (Delgado et al., 2018). Strains of antibiotic-sensitive *P. aeruginosa* (strain PaS), multi-resistant *P. aeruginosa* (strain PaR), and SMG were used in the subsequent experiments. The PaS strain is sensitive to ceftazidime, ciprofloxacin, gentamicin, amikacin, imipenem, meropenem, piperacillin/tazobactam, and colistine, while PaR is resistant to all the mentioned antibiotics except for colistine (Delgado et al., 2018).

In addition, the *P. aeruginosa* 101 strain, a qsc mutant that was a generous gift from E.P. Greenberg and K. Lee from the University of Iowa, USA, was used (Whiteley et al., 1999). The qsc mutant has random *lacZ* transcriptional fusions in the chromosome of a *las*I–*rhl*I double mutant and was used to detect the production of AHL by wild-type *P. aeruginosa* strains. The qsc mutant strain does not produce either the autoinducer N-3-oxododecanoyl homoserine lactone (3O-C12-HSL) or the butanoyl-L-homoserine lactone (C4-HSL) but responds to the presence of these compounds by increasing the expression of β-galactosidase. *P. aeruginosa* 101, referred to here as Paqsc, was selectively grown in an LB medium containing gentamicin 100 mg/L (Whiteley et al., 1999). Colonies of the Paqsc strain were then cultured in LB broth without antibiotics.

### Bacterial supernatants

*P. aeruginosa* strains were cultured in LB broth, while SMG were cultured in BHI medium. After overnight growth at 37°C, these cultures were used to obtain the supernatants of each strain by centrifugation for 30 min at 30,000 *g* at 10°C and the filtration of the supernatant through a millipore filter of 0.22 μm in diameter. These supernatants were kept in a refrigerator at 4°C until use. *L. plantarum* ATCC^®^ 10241^TM^ strain was cultured in MRS broth (Britania, Argentina) at 37°C. The supernatant from lactobacilli culture was obtained as described previously (Peral et al., 2010) and stored at 4°C.

### Biofilm formation

A static biofilm assay was performed as described elsewhere (O'Toole and Kolter, 1998). A total of 100 μl/well (10^8^ CFU) of the overnight culture of the different *P. aeruginosa* strains (PaS, PaR, or Paqsc) were placed in 96 well plates. Then, 100 μl of the supernatant from *P. aeruginosa* (PaS, PaR, or Paqsc), SMG, or 100 μl of cultures of the same microorganisms were added. To determine the effect of the supernatant of *L. plantarum* on biofilm formation and viability of *P. aeruginosa* strains (PaS, PaR, or Paqsc) we followed the procedure described: a total of 100 μl of *P. aeruginosa* strains cultures were added to each well, and then 50 μl of the supernatant from the lactic acid bacterium grown in BHI, LB, or MRS media. As a control, 100 μl of the culture media for each bacteria was used. The plates were incubated for 6 h at 37°C and then stained with crystal violet. The cell-attached dye was solubilized with ethanol at 95% v/v, and the absorbance was then measured at 540 nm. All assays were performed in triplicate.

### Bacterial viability

The viability of bacteria in biofilms alone and the planktonic plus biofilm states was performed according to the assay described by Cerca et al. (2005). A total of 100 μl of overnight culture of the different *P. aeruginosa* strains (PaS, PaR, or Paqsc; 10^8^ CFU) were placed in 96-well plates. Then, 50 μl of the supernatants of *L. plantarum*, SMG, or mixed supernatants were added. Wells with 100 μl of *P. aeruginosa* strain cultures in 100 μl of BHI medium were used as controls.

To determine the viability of bacteria within the biofilm, the wells were emptied and washed four times with PBS to remove planktonic cells. Then, they were refilled with 200 μl of PBS. Then, 50 μl of XTT salt solution (1 mg/ml in PBS; Promega, Madison, WI, USA) was added to each well and incubated at 37°C in the dark for 90 min. Bacterial dehydrogenase activity reduces XTT tetrazolium salt to XTT formazan, resulting in a colorimetric change that can be measured spectrophotometrically at 490 nm, which correlates with cell viability (Valdéz et al., 2005).

### Production of AHL

To investigate the production of AHL by *P. aeruginosa* strains (PaS, PaR, or Paqsc), bacteria were grown to an OD600 of 0.7, centrifuged, and filtered. In a hemolysis tube, 500 μl of each filtrate was added to 500 μl of the Paqsc strain culture. Following incubation at 37°C for 1 h, β-galactosidase activity was measured by the Miller reaction, and the results were expressed in β-galactosidase units (Miller, 1992).

### Animal model of lung infection

A model of lung infection with *P. aeruginosa* using BALB/c mice was developed following previous studies (Delgado et al., 2018; Brao et al., 2020). This model uses an intranasal route of infection and does not embed bacteria in foreign substances to prevent clearance, resembling a natural infection and allowing the study of *P. aeruginosa* in a mouse with CF-like lung disease for up to 12 days post-infection. The Institutional Animal Care and Use Committee at the University of Maryland, Baltimore, MD, approved the experiments, and it was endorsed by the Bioethics Committee of the Faculty of Biochemistry, Chemistry and Pharmacy, National University of Tucuman (Tucuman, Argentina).

Adult BALB/c mice (6 weeks) from the random-bred colony kept in the Department of Immunology at the Institute of Microbiology of the Tucuman University (Tucuman, Argentina) were randomized in experimental groups of six mice each. Mice were housed with food and water *ad libitum* and a 12 h light/dark cycle. Animals were anesthetized by intraperitoneal injection of ketamine hydrochloride (75 mg/kg) and medetomidine hydrochloride (1 mg/kg) to be infected or sacrificed.

**Figure 1:**
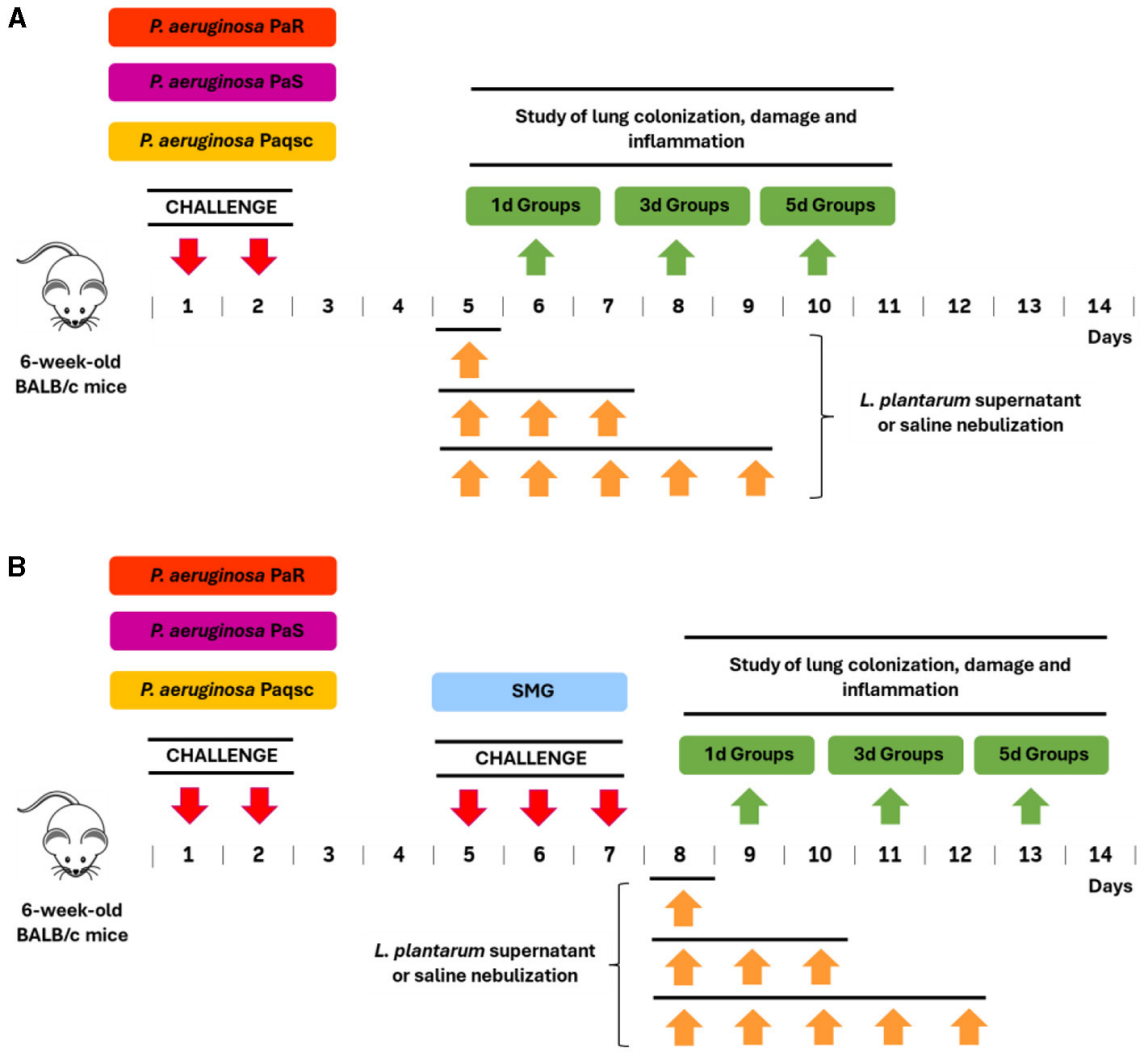
Experimental protocols were used to evaluate the effect of *L. plantarum* supernatant on respiratory infections produced by CF-derived pathogens. **(A)** The effects of *L. plantarum* supernatant on respiratory infections produced by different *P. aeruginosa* strains (Paqsc, PaS, and PaR) were evaluated 5 days after the challenges with the pathogens and for 1, 3, or 5 days through nebulization. **(B)** The effect of *L. plantarum* supernatant on respiratory superinfections produced by different *P. aeruginosa* strains (Paqsc, PaS, and PaR) and the *Streptococcus milleri* group (SMG) was evaluated 1 day after the challenge with SMG and for 1, 3, or 5 days through nebulization. Mice nebulized with saline solution were used as controls.

To produce lung infection, mice were instilled intranasally with 50 μl of *P. aeruginosa* strains (PaS, PaR, or Paqsc) suspensions (10^6^ CFU) for two consecutive days. In this schedule, mice infected with PaS, PaR, or Paqsc strains displayed signs of disease in the first 3 days of infection, including ruffled fur and hunched posture. After 6, 8, or 10 days, mice were anesthetized and sacrificed to obtain samples of bronchoalveolar lavages (BAL) and lungs for quantitative bacteriology (Figure 1A).

For bacterial co-infection experiments, mice were intranasally challenged with different *P. aeruginosa* strains (PaS, PaR, or Paqsc) for 2 consecutive days as described above and, 5 days later, infected with the 25 μl of SMG (2.5 × 10^4^ CFU) for 3 consecutive days (Figure 1B). Control groups were mice instilled only with *P. aeruginosa* strains or saline. Moreover, 1, 3, or 5 days after the last SMG challenge (days 9, 11, and 13), animals were anesthetized and sacrificed to obtain samples of BAL and lungs for quantitative bacteriology and histopathology lung studies (Figure 1B).

### *L. plantarum* treatment

Mice infected with the different *P. aeruginosa* strains (PaS, PaR, or Paqsc) were nebulized with *L. plantarum* culture supernatant from day 5 and for 1, 3, or 5 days (Figure 1A). Mice infected with *P. aeruginosa* strains and coinfected with SMG were nebulized with *L. plantarum* culture supernatant from day 8 and for 1, 3, or 5 days (Figure 1B). Control groups were mice instilled with saline during *P. aeruginosa* or SMG challenges and nebulized with *L. plantarum* supernatant. Nebulizations were performed for 5 min per day. The nebulization with *L. plantarum* culture supernatant and saline was conducted by a compressor/nebulizer (OMROM NE-C801) connected by a tube to a hermetically sealed plastic container. The mice to be nebulized were placed into this container.

### Quantitative lung bacteriology

To determine bacterial cell counts in the respiratory tract of infected mice, the whole lungs of animals were aseptically excised, weighed, and homogenized in 5 ml of sterile 0.9% saline. Serial dilutions were immediately plated on Levin, BCSA agar, and Sodium Azide agar (Biomeriex, Argentina), and the numbers of viable bacteria were determined following overnight incubation at 37°C. The different culture mediums were used because they are selective for the pathogens studied (Sibley et al., 2008).

### Studies in BAL

BAL samples were collected as described before (Tonetti et al., 2023). Briefly, 0.5 ml of saline containing 10 U/ml of heparin was injected and aspirated thrice through the tracheostomy tube. The total volume of BAL fluid was pooled. Then, BAL cells were counted in a hemocytometer, and the differential cell types were determined by smears stained with Giemsa. Hemoglobin was determined by spectrophotometry. The determination of elastase activity was carried out following the procedures of Gambello and Iglewski (1991). Briefly, 500 μl of elastin-congo red solution (20 mg of congo red elastin in 1 ml of 10 mM Na_2_HPO_4_, pH = 7) was used as a substrate. Samples (500 μl of BAL) were incubated overnight with shaking for 24 h at 37°C. The pellet was removed by centrifugation, and the absorbance of the supernatant was read on a spectrophotometer at OD 495 nm.

### Histopathology

After the sacrifice of mice, the lungs were instilled with 1 ml of 10% formaldehyde using a tracheal catheter. The whole lung was removed and processed for routine histological hematoxylin-eosin staining. The degree of edema, necrosis, hemorrhage, congestion, alveolar inflammatory infiltrate, and interstitial inflammatory infiltrate was scored on scales from 0 to +++: absence (0), mild focal (+), moderate to severe focal (++), and severe throughout the lung (+++). Histopathology evaluation was performed blindly.

### Statistical analysis

All assays were performed at least in triplicate, and the results were expressed as mean values with standard deviations. Since the lungs of infected mice were used for histology, quantitative bacteriology, and the evaluation of parameters in BAL samples, and it is not possible to perform all these determinations in the same lung, different experiments were performed in each case. The number of repetitions is incorporated into each figure. Statistical analyses were performed using GraphPad Prism 8 software (La Jolla, CA, USA), and a *t*-test (for pairwise comparisons of the means) was used to test for differences between the groups. Differences were considered significant at a *p*-value of <0.05.

## Results

### Effect of *L. plantarum* supernatant on *P. aeruginosa* biofilm formation *in vitro*

**Figure 2:**
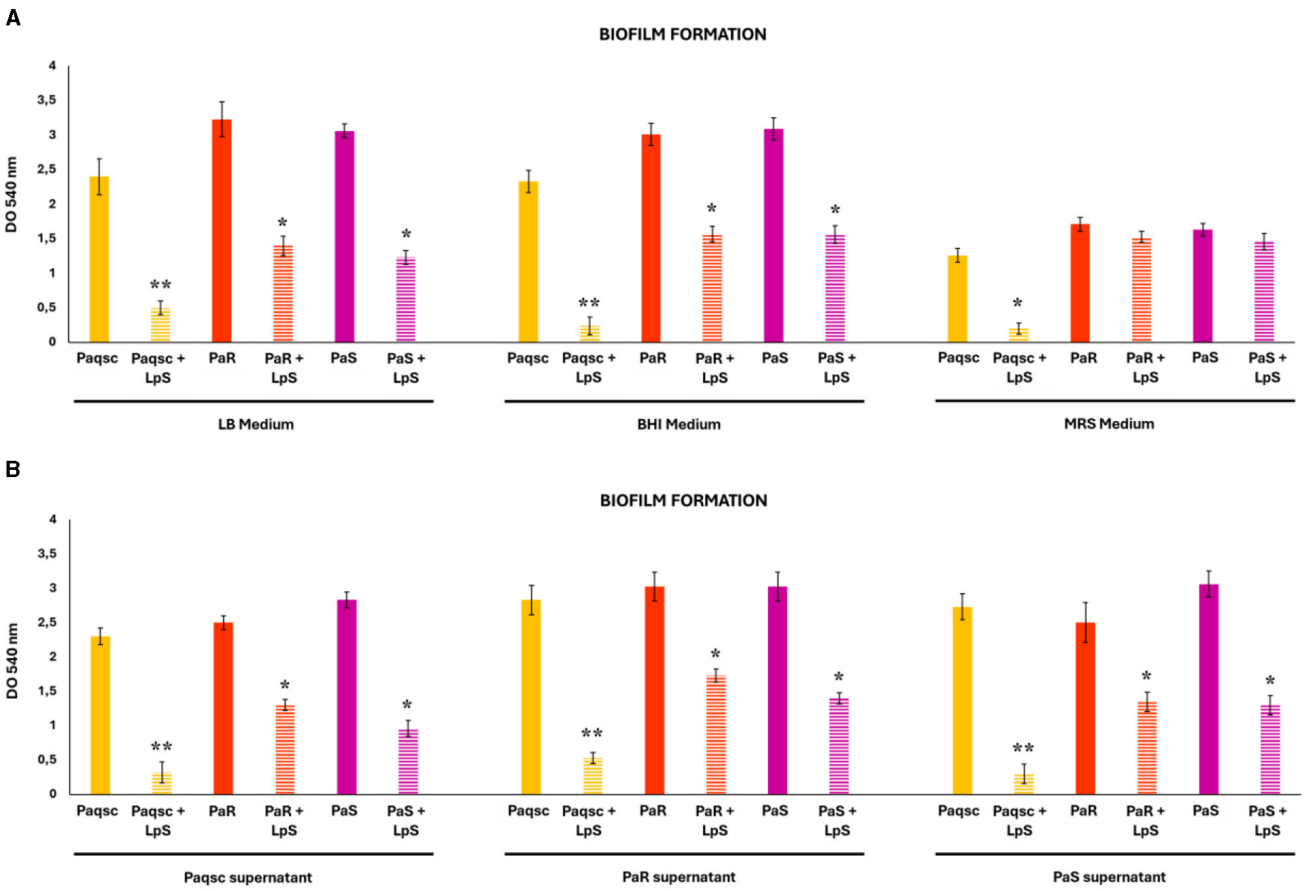
Effect of *L. plantarum* supernatants on *P. aeruginosa* biofilms. **(A)** Different *P. aeruginosa* strains (PaR, PaS, and Paqsc) were grown in LB, BHI, and MRS mediums, and the absorbance values (OD540) obtained from the cultures using the crystal violet method were used to evaluate biofilms. **(B)** The effects of *L. plantarum* and *P. aeruginosa* strain-conditioned mediums on biofilm formation are also shown. Data represent the mean ± SD of triplicated experiments (*n* = 3 per experiment). Asterisks show statistical significance between *L. plantarum* treated and the respective control groups without lactobacilli treatment *p < 0.05 and **p < 0.01.

The ability of three *P. aeruginosa* strains (PaR, PaS, and Paqsc) to form biofilms and the effect of *L. plantarum* supernatant were evaluated in different culture mediums (Table 1, Figure 2), taking into consideration that the metabolites produced by the bacteria that are involved in the antimicrobial activities depend on the medium that they grow (Valdéz et al., 2005; Ramos et al., 2015). The cultures of *P. aeruginosa* strains in the LB and BHI mediums showed significantly higher biofilm production than in the MRS medium. It was observed that PaR and PaS did not have significant differences in biofilm formation between them, and both showed greater biofilm production capacity than the Paqcs strain (Table 1, Figure 2). These differences were observed in the three mediums evaluated. The addition of culture supernatants from PaS and Paqsc strains to *P. aeruginosa* PaR culture in LB medium reduced the production of biofilm, while supernatants from PaR and Paqsc strains to *P. aeruginosa* PaS culture did not affect the biofilm production (Table 1, Figure 2). It was also observed a tendency for higher levels of biofilm production in the Paqsc cultures treated with the supernatants of the PaR and PaS strains. However, the differences were not statistically significant.

**Table 1:** Effect of *L. plantarum* supernatants on *P. aeruginosa* biofilms.

***P. aeruginosa* strains**	**Medium**			***P. aeruginosa*** **strains supernatants**		
	**LB**	**BHI**	**MRS**	**PaR**	**PaS**	**Paqsc**
PaR	3.23 ± 0.25	3.00 ± 0.16	1.70 ± 0.08^**^	3.03 ± 0.21	2.50 ± 0.29^*^	2.50 ± 0.08^*^
PaR + *L. plantarum* supernatant	1.40 ± 0.14^†^	1.57 ± 0.11^†^	1.50 ± 0.008	1.37 ± 0.047^†^	1.35 ± 0.14^†^	1.30 ± 0.005^†^
PaS	3.06 ± 0.26	3.09 ± 0.16	1.63 ± 0.05^*^	3.03 ± 0.21	3.06 ± 0.19	2.83 ± 0.12
PaS + *L. plantarum* supernatant	1.23 ± 0.09^†^	1.56 ± 0.13^†^	1.46 ± 0.12	1.40 ± 0.05^†^	1.30 ± 0.14^†^	0.96 ± 0.12^†^
Paqsc	2.40 ± 0.26	2.33 ± 0.16	1.26 ± 0.05^*^	2.83 ± 0.21	2.73 ± 0.19	2.30 ± 0.12
Paqsc + *L. plantarum* supernatant	0.50 ± 0.09^†^	0.24 ± 0.13^†^	0.20 ± 0.12^†^	0.53 ± 0.05^†^	0.30 ± 0.14^†^	0.32 ± 0.15^†^

Different *P. aeruginosa* strains (PaR, PaS, and Paqsc) were grown in LB, BHI, and MRS mediums and the absorbance values (OD540) obtained from the cultures using the crystal violet method were used to evaluate biofilms. The effects of *L. plantarum* and *P. aeruginosa* strains conditioned mediums on biofilm formation are also shown. Data represent the mean ± SD of triplicated experiments. Asterisks show statistical significance between the indicated groups and *P. aeruginosa* strains grown in LB medium in the same row, ^*^p <0.05 and ^**^p <0.01. Symbols show statistical significance between *L. plantarum* treated and the respective control groups without lactobacilli treatment in the same column, ^†^p <0.05.

Interestingly, when cultures of *P. aeruginosa* strains were performed in the presence of *L. plantarum* supernatant, a significant inhibition of biofilm formation was observed for the three strains in both LB and BHI mediums (Figure 2). The reduction of biofilm formation in the MRS medium by *L. plantarum* supernatant was only detected for the Paqsc strain. The cell-free supernatant of *L. plantarum* was also capable of reducing biofilm formation of the three *P. aeruginosa* strains in the experiments in which the bacteria were cultured in the presence of the supernatants of the PaR, PaS, and Paqsc strains (Figure 2). The viability of *P. aeruginosa* strains within the biofilms was also evaluated (Table 2). The three *P. aeruginosa* strains showed higher viability in LB and BHI than in the MRS medium. In addition, it was detected that *L. plantarum* supernatant significantly reduced the viability of the PaR, PaS, and Paqsc strains within the biofilm produced in the LB medium (Table 2).

**Table 2:** Effect of *L. plantarum* supernatants on *P. aeruginosa* viability.

***P. aeruginosa* strains**	**Medium**			
	**LB**	**BHI**	**MRS**	**LB** + ***L. plantarum*** **supernatant**
PaR	0.48 ± 0.05	0.48 ± 0.11	0.22 ± 0.07^*^	0.21 ± 0.05^*^
PaS	0.47 ± 0.08	0.40 ± 0.07	0.30 ± 0.07^*^	0.18 ± 0.08^*^
Paqsc	0.38 ± 0.05	0.37 ± 0.05	0.16 ± 0.03^*^	0.12 ± 0.05^*^

Different *P. aeruginosa* strains (PaR, PaS, and Paqsc) were grown in LB, BHI, and MRS mediums and the absorbance values (OD450) obtained from the cultures using the XTT reduction method were used to evaluate viability within the biofilm. The effects of *L. plantarum* conditioned medium on viability within the biofilms are also shown. Data represent the mean ± SD of triplicated experiments. Asterisks show statistical significance between the indicated groups and *P. aeruginosa* strains grown in LB medium in the same row, ^*^p <0.05.

**Figure 3:**
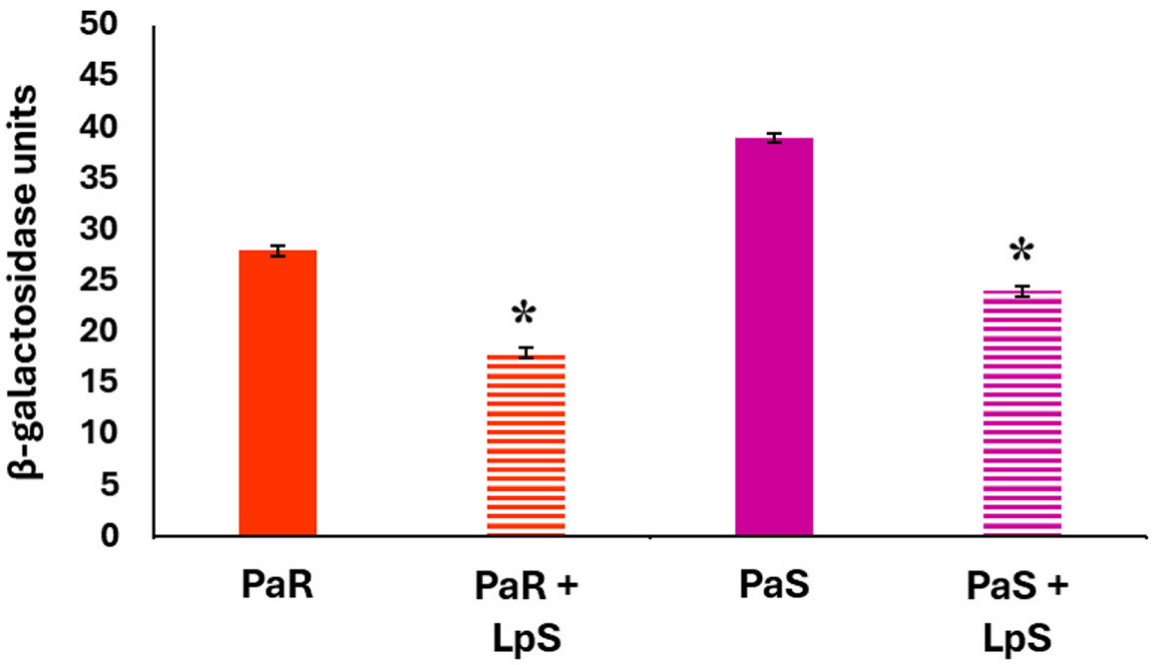
Effect of *L. plantarum* supernatant on *P. aeruginosa* AHL production. The effects of *L. plantarum* supernatant on *P. aeruginosa* strains (PaR, PaS) acyl-homoserine lactone (AHL) production are shown. AHL production is measured as β-galactosidase units. Data represent the mean ± SD of triplicated experiments (*n* = 3 per experiment). Asterisks show statistical significance between *L. plantarum* treated and the respective control groups (PaR and PaS) without lactobacilli treatment, *p < 0.05.

We also evaluated AHL production in *P. aeruginosa* PaR and PaS cultures by measuring β-galactosidase activity as described in materials and methods (Figure 3). The study of the AHL production revealed that the PaS strain (UE = 38.71) had a higher activity than *P. aeruginosa* PaR (UE = 26.54). The cell-free supernatant of *L. plantarum* could also reduce the AHL production in both *P. aeruginosa* PaR and PaS cultures (Figure 3).

### Effect of *L. plantarum* supernatant on *P. aeruginosa* respiratory infection *in vivo*

**Figure 4:**
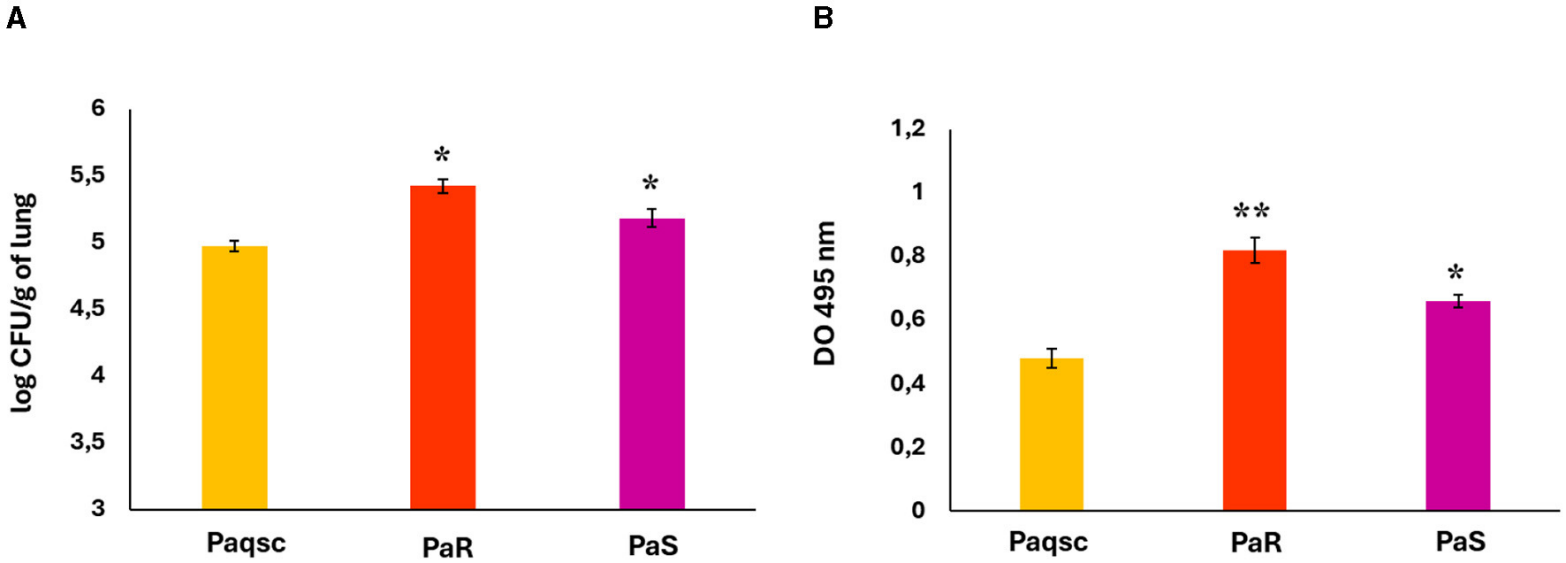
Abilities of *P. aeruginosa* strains to produce respiratory infections. Mice were nasally challenged with different *P. aeruginosa* strains (PaR, PaS, and Paqsc) and **(A)** the bacterial counts in the lungs and **(B)** the elastase activity in bronchoalveolar lavages (BAL) samples were determined. Data represent the mean ± SD of triplicated experiments (*n* = 3 per experiment). Asterisks show statistical significance compared to Paqsc, *p < 0.05, **p < 0.01.

Considering the ability of *L. plantarum* supernatant to affect the viability and biofilm production of the three *P. aeruginosa* strains, we next aimed to evaluate whether this supernatant could influence the outcome of respiratory infections caused by opportunistic pathogens. Then, we first assessed the abilities of PaR, PaS, and Paqsc to colonize the lungs of mice nasally challenged with the bacteria (Figure 4A). It was observed that the three *P. aeruginosa* strains were able to colonize the lower respiratory tract of mice. However, the lung bacterial cell counts in mice challenged with PaR and PaS were significantly higher than those observed in the group infected with the Paqsc strain. The elastase release in BAL samples was also determined to indirectly measure *P. aeruginosa* strain virulence. Elastase is under the detection limit in BAL samples of non-infected mice, while the challenges with PaR, PaS, or Paqsc significantly increased the values of this parameter in infected animals. The elastase release in mice challenged with *P. aeruginosa* Paqsc was significantly lower than observed in the groups infected with PaR or PaS strains (Figure 4B). The results indicate that PaR and PaS would be more virulent than the mutant Paqsc strain that is not able to produce AHL, an important pathogenicity factor. Furthermore, elastase release was higher in the BAL of animals infected with PaR than those challenged with *P. aeruginosa* PaS (Figure 4B).

**Figure 5:**
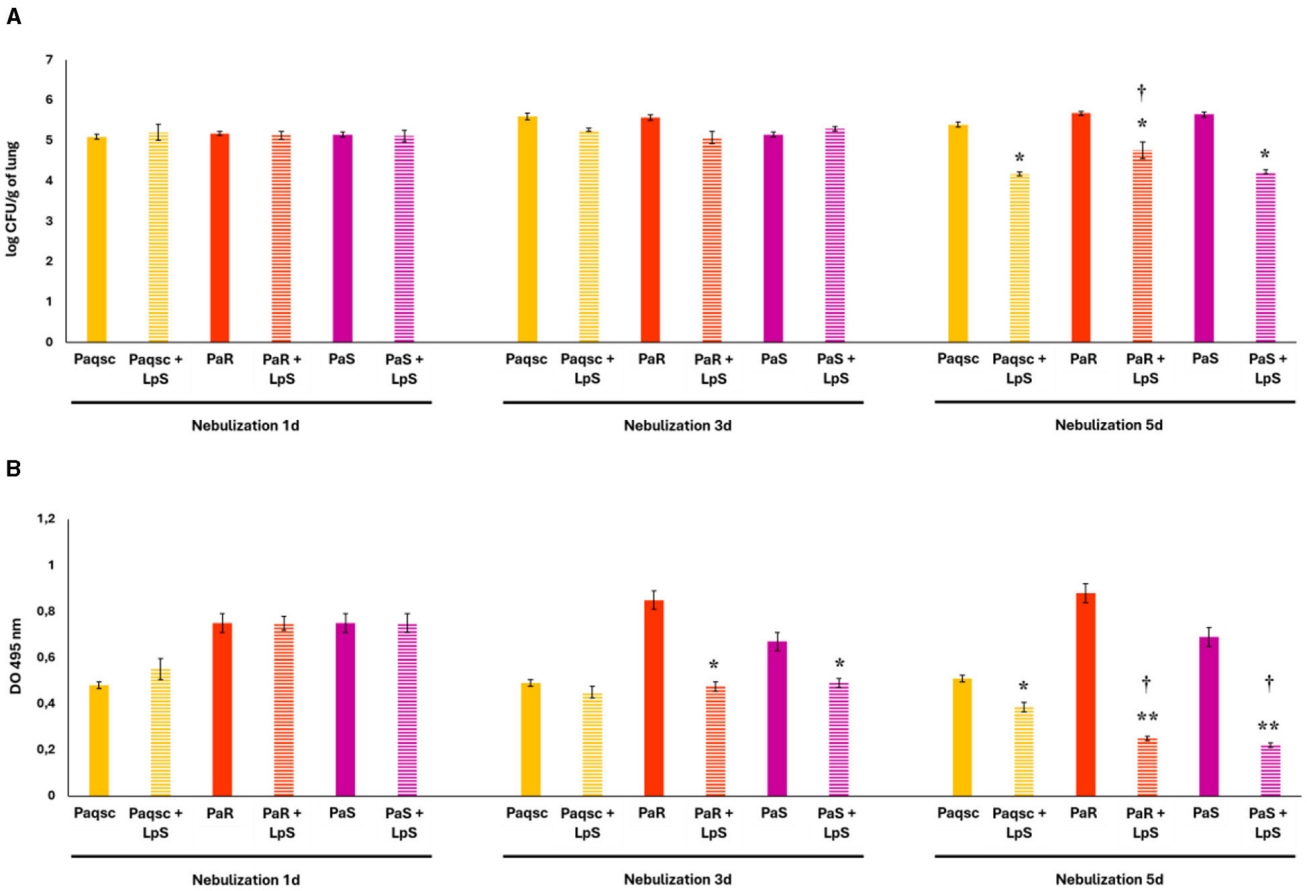
Effect of *L. plantarum* supernatant on respiratory infections produced by *P. aeruginosa* strains. The effects of *L. plantarum* supernatant on respiratory infections produced by different *P. aeruginosa* strains (Paqsc, PaS, and PaR) are shown. **(A)** The bacterial counts in the lungs and **(B)** elastase release in bronchoalveolar lavages (BAL) were determined. Data represent the mean ± SD of triplicated experiments (*n* = 3 per experiment). Asterisks show statistical significance compared to the respective non-supernatant-treated control, *p < 0.05, **p < 0.01. Symbols show statistical significance compared with the Paqsc group, ^†^*p* < 0.05.

The effect of *L. plantarum* supernatant on *P. aeruginosa* respiratory infections was evaluated after 1, 3, and 5 days of nebulizations (Figure 5). Lung bacterial cell counts after the nebulizations with *L. plantarum* supernatant for 1 or 3 days were not different from controls for strains PaR, PaS, and Paqsc (Figure 5A). In contrast, *L. plantarum* supernatant significantly reduced lung bacterial cell counts after 5 days of nebulizations in the three groups of mice infected with the *P. aeruginosa* strains (Figure 5A). When bacterial loads in the lower respiratory tract of infected mice were compared, no differences were detected between the strains after 3 days of nebulization with *L. plantarum* supernatant. In contrast, lung bacterial cell counts in mice infected with *P. aeruginosa* PaR and treated with *L. plantarum* supernatant for 5 days were significantly higher than the values detected in animals infected with PaS and Paqsc strains (Figure 5A).

The effect of *L. plantarum* supernatant on elastase release in BAL samples was also determined for the three *P. aeruginosa* strains (Figure 5B). The nebulization of mice infected with PaR and PaS with *L. plantarum* supernatant significantly reduced the levels of elastase release in BAL after 3 and 5 days, making the effect more remarkable on day 5. The elastase release in BAL samples of mice infected with *P. aeruginosa* Paqsc and treated with *L. plantarum* supernatant differed from control only at day 5 (Figure 5B).

We also evaluated the number of leukocytes in BAL samples to measure the inflammatory response (Table 3). The challenge of mice with *P. aeruginosa* strains significantly increased the number of BAL leukocytes compared to basal levels, the most remarkable effect observed in animals infected with the PaR strain. The treatment with *L. plantarum* supernatant significantly reduced the levels of BAL leukocytes after 1, 3, and 5 days for the three *P. aeruginosa* strains. The lowest number of leucocytes in mice infected with PaR, PaS, and Paqsc strains and treated with *L. plantarum* supernatant were observed at day 5 (Table 3). However, these values did not reach the numbers observed in non-infected animals. When the different types of leukocytes were analyzed in BAL samples, it was observed that in mice infected with *P. aeruginosa* Paqsc, 90% corresponded to neutrophils, 5% to macrophages, and 5% to lymphocytes. In animals challenged with the PaS strains, the BAL leukocytes were composed of 30% of neutrophils and 70% of macrophages, while in PaR-infected mice, the composition was 5% of neutrophils and 95% of macrophages (Supplementary Figure 1).

**Table 3:** Effect of *L. plantarum* supernatant on respiratory infections produced by *P. aeruginosa* strains.

***P. aeruginosa* strains**	**BAL leukocytes**			
	**No treatment**	***L. plantarum*** **supernatant 1d**	***L. plantarum*** **supernatant 3d**	***L. plantarum*** **supernatant 5d**
PBS	2.5 × 10^1^ ± 0.4	-	-	-
PaR	5.3 × 10^3^ ± 0.5^†^	1.6 × 10^3^ ± 0.3^*^	1.5 × 10^3^ ± 0.3^*^	1.3 × 10^3^ ± 0.2^*^
PaS	4.4 × 10^3^ ± 0.6^†^	1.1 × 10^3^ ± 0.4^*^	9.6 × 10^2^ ± 0.3^*^	1.9 × 10^2^ ± 0.4^*^
Paqsc	3.3 × 10^3^ ± 0.5^†^	1.2 × 10^2^ ± 0.4^*^	8.6 × 10^2^ ± 0.2^*^	1.2 × 10^2^ ± 0.3^*^

Leukocytes counts in BAL samples. Data represent the mean ± SD of triplicated experiments. Asterisks show statistical significance between the indicated groups and non-lactobacilli treated controls in the same row, ^*^p <0.05. Symbols show statistical significance between the indicated groups and controls in the same column, ^†^p <0.05.

**Figure 6:**
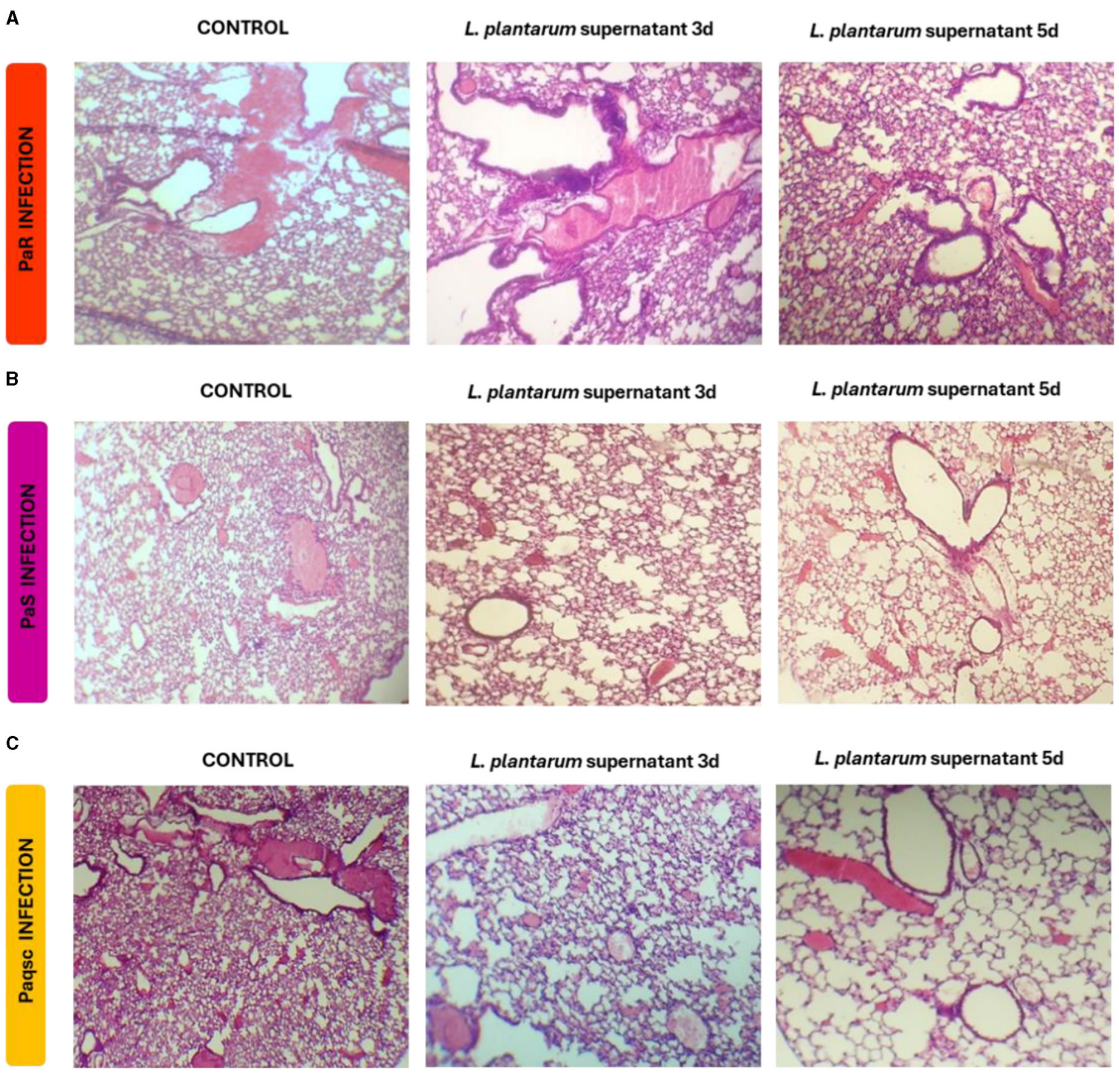
Effect of *L. plantarum* supernatant on respiratory infections produced by *P. aeruginosa* strains. The effects of *L. plantarum* supernatant on respiratory infections produced by different *P. aeruginosa* strains (Paqsc, PaS, and PaR) are shown. The histology of the lungs was determined. **(A–C)** Lung histopathological analysis at different points post-infection and treatment with *L. plantarum* supernatant for PaR, PaS, and Paqsc, respectively. Data were obtained from duplicated experiments (*n* = 3 per experiment).

Lung histology was also studied to evaluate the tissue damage induced by *P. aeruginosa* infections (Figure 6 and Table 4). The three *P. aeruginosa* strains could induce lung tissue injury compared to non-infected controls (Supplementary Figure 2), although with a different degree of severity. The PaR strain induced hemorrhage, edema, congestion, and inflammatory infiltrates both in the alveolar and the interstitial compartments (Figure 6A). Animals challenged with *P. aeruginosa* PaS showed edema, moderate congestion, hemorrhage, and scant inflammatory infiltrate in the lung interstitial tissue (Figure 6B). The histopathological analysis also showed that the infection produced by the mutant Paqsc strain was much milder when compared to the strains of clinical origin, PaR and PaS (Figure 6 and Table 4). When the effect of *L. plantarum* supernatant was evaluated, it was determined that after 1 or 3 days of treatments, there were no changes in lung tissue damage compared to the respective controls for any of the *P. aeruginosa* strains, as shown in the microscopic observations (Figure 6) and the scores (Table 4). However, the nebulization with *L. plantarum* supernatant for 5 days significantly decreased lung damage in animals infected with any of the three *P. aeruginosa* strains.

**Table 4:** Effect of *L. plantarum* supernatant on respiratory infections produced by *P. aeruginosa* strains.

**Treatment**	**Edema**	**Necrosis**	**Hemorrhage**	**Congestion**	**Alveolar inflammatory infiltrate**	**Interstitial inflammatory infiltrate**
PaR	++	+	++	++	+	++
PaR + *L. plantarum* supernatant 1d	++	+	++	++	+	++
PaR + *L. plantarum* supernatant 3d	++	0	++	+	0	++
PaR + *L. plantarum* supernatant 5d	+	0	0	0	0	+
PaS	+++	0	+	+	0	+
PaS + *L. plantarum* supernatant 1d	+++	0	+	+	0	+
PaS + *L. plantarum* supernatant 3d	++	0	+	+	0	+
PaS + *L. plantarum* supernatant 5d	+	0	0	+	0	0
Paqsc	+	0	+	+	0	+
Paqsc + *L. plantarum* supernatant 1d	+	0	+	+	0	+
Paqsc + *L. plantarum* supernatant 3d	+	0	+	+	0	+
Paqsc + *L. plantarum* supernatant 5d	+	0	0	0	0	0

Histopathological score of lung samples. The effects of *L. plantarum* supernatant on respiratory infections produced by different *P. aeruginosa* strains (Paqsc, PaS, and PaR) are shown. Lung histopathological analysis at different points post-infection and treatment with *L. plantarum* supernatant was determined. The degree of edema, necrosis, hemorrhage, congestion, alveolar inflammatory infiltrate, and interstitial inflammatory infiltrate was scored on scales from 0 to +++: absence (0), mild focal (+), moderate to severe focal (++), and severe throughout the lung (+++). Data were obtained from duplicated experiments (*n* = 3 per experiment).

### Effect of *L. plantarum* supernatant on *P. aeruginosa* and SMG biofilm formation *in vitro*

Subsequently, we aimed to evaluate the interaction of the three *P. aeruginosa* strains (PaR, PaS, and Paqsc) with SGM and the potential beneficial effect of *L. plantarum* supernatant on that interaction. The effect of SMG and SMG supernatants in the ability to form biofilms was evaluated in different culture mediums (Table 5). Both SMG and SMG supernatants significantly reduced the capacity of *P. aeruginosa* strains PaR, PaS, and Paqsc to form biofilms. The cell-free supernatant of *L. plantarum* could also reduce the biofilm formation of the three *P. aeruginosa* strains cocultured with SMG or SMG supernatants (Table 5).

**Table 5:** Effect of *L. plantarum* supernatants on *P. aeruginosa* and SMG biofilms.

***P. aeruginosa* strains**	**LB**	**SMG supernatants**	**SMG**
PaR	3.23 ± 0.25	2.50 ± 0.08^*^	2.58 ± 0.06^*^
PaR + *L. plantarum* supernatant	1.40 ± 0.14^†^	1.33 ± 005^†^	1.32 ± 0.12^†^
PaS	3.06 ± 0.26	2.23 ± 0.12^*^	2.35 ± 0.15^*^
PaS + *L. plantarum* supernatant	1.23 ± 0.09^†^	1.33 ± 0.04^†^	1.35 ± 0.12^†^
Paqsc	2.40 ± 0.26	1.43 ± 0.12^*^	1.33 ± 0.15^*^
Paqsc + *L. plantarum* supernatant	0.50 ± 0.09^†^	0.17 ± 0.05^†^	0.33 ± 0.14

Different *P. aeruginosa* strains (PaR, PaS, and Paqsc) were grown in LB mediums with the addition of SMG or SMG supernatants and the absorbance values (OD450) obtained from the cultures using the crystal violet method were used to evaluate biofilms. The effects of *L. plantarum* conditioned medium on biofilm formation are also shown. Data represent the mean ± SD of triplicated experiments. Asterisks show statistical significance between the indicated groups in the same row, ^*^p <0.05. Symbols show statistical significance between *L. plantarum* treated and the respective control groups without lactobacilli treatment in the same column, ^†^p <0.05.

The viability of *P. aeruginosa* strains within the biofilms in coculture with SMG supernatant was also evaluated (Table 6). When the SMG supernatant was added, the three *P. aeruginosa* strains showed lower viability in biofilms. In addition, it was observed that *L. plantarum* supernatant significantly reduced the viability of the PaR, PaS, and Paqsc strains within the biofilm produced in LB medium coculture with SMG supernatant (Table 6).

**Table 6:** Effect of *L. plantarum* and SMG supernatants on *P. aeruginosa* viability.

***P. aeruginosa* strains**	**Medium**		
	**LB**	**LB** + **SMG supernatant**	**LB** + **SMG** + ***L. plantarum*** **supernatant**
PaR	0.48 ± 0.05	0.38 ± 0.07^*^	0.20 ± 0.07^**^
PaS	0.47 ± 0.08	0.37 ± 0.06^*^	0.18 ± 0.08^**^
Paqsc	0.38 ± 0.05	0.25 ± 0.06^*^	0.12 ± 0.03^*^

Different *P. aeruginosa* strains (PaR, PaS, and Paqsc) were grown in LB medium and the absorbance values (OD450) obtained from the cultures using the XTT reduction method were used to evaluate viability within the biofilm. The effects of *L. plantarum* and SMG conditioned medium on viability within the biofilms are also shown. Data represent the mean ± SD of triplicated experiments. Asterisks show statistical significance between the indicated groups and *P. aeruginosa* strains grown in LB medium in the same row, ^*^p <0.05 and ^**^p <0.01.

### Effect of *L. plantarum* supernatant on *P. aeruginosa* and SMG respiratory infection *in vivo*

**Figure 7:**
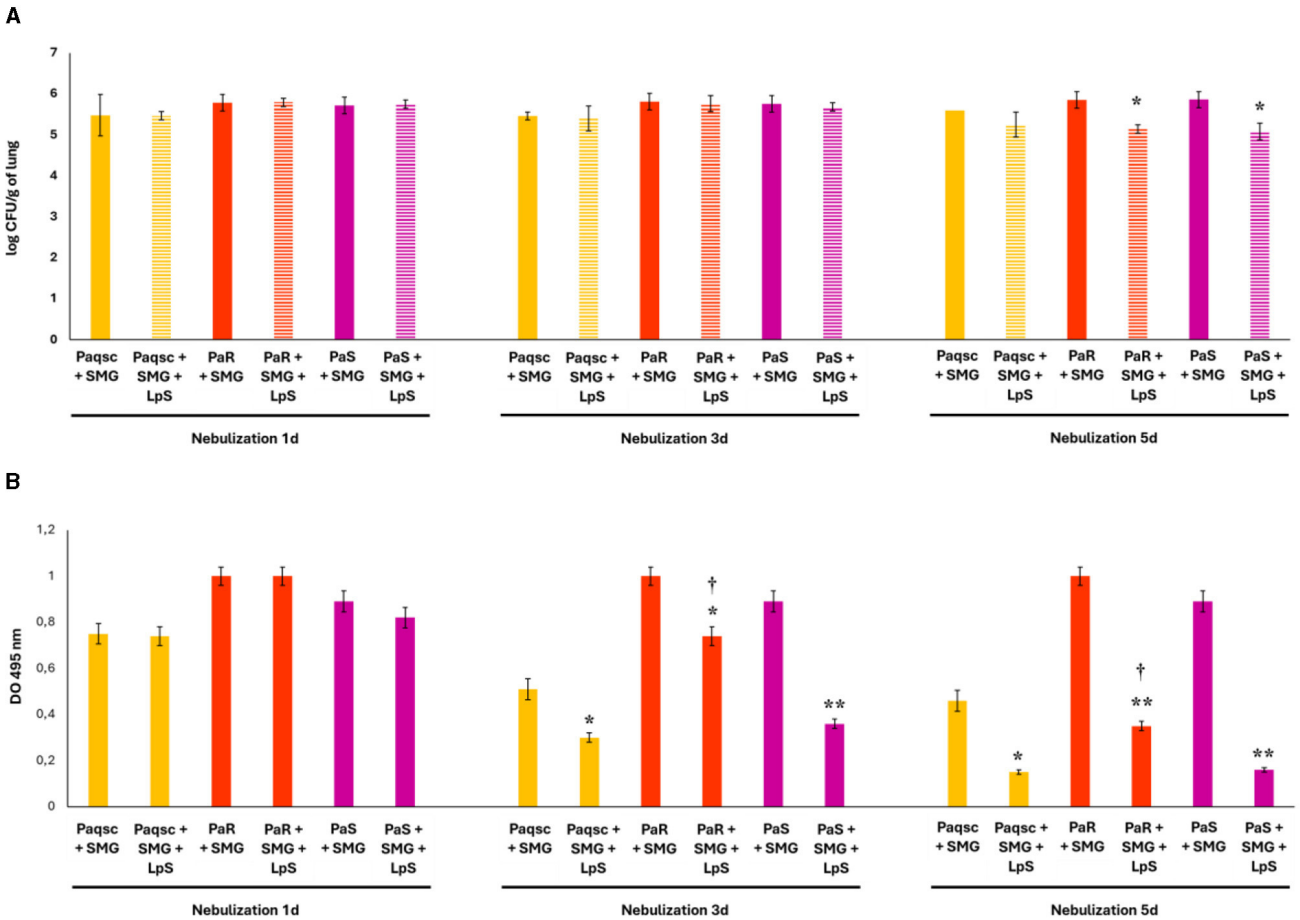
Effect of *L. plantarum* supernatant on respiratory superinfections produced by *P. aeruginosa* strains and SMG. The effects of *L. plantarum* supernatant on respiratory superinfections produced by different *P. aeruginosa* strains (Paqsc, PaS, and PaR) and SMG are shown. **(A)** The bacterial counts in the lungs and **(B)** elastase release in bronchoalveolar lavages (BAL) were determined. Data represent the mean ± SD of triplicated experiments (*n* = 3 per experiment). Asterisks show statistical significance compared to the respective non-supernatant-treated control, *p < 0.05, **p < 0.01. Symbols show statistical significance compared with the Paqsc group, ^†^*p* < 0.05.

Considering the ability of *L. plantarum* supernatant to affect the viability and biofilm production of the three *P. aeruginosa* strains in coculture with SMG supernatant, we next aimed to evaluate whether this supernatant could be used to influence the outcome of respiratory infections caused by *P. aeruginosa* and SMG. Then, we evaluated the abilities of PaR, PaS, and Paqsc to colonize the lungs of mice nasally challenged with the bacteria and infected 5 days later with SMG (Figure 7A). The lung bacterial counts for the three strains in experiments with SMG challenge were slightly higher than that observed when *P. aeruginosa* was used alone: PaR 5.8 vs. 5.4 log CUF/g of the lung and PaS 5.7 vs. 5.3 log CUF/g of the lung (Figure 7A). However, the lung bacterial cell counts in mice challenged with PaR and PaS were significantly higher than those observed in the group infected with the Paqsc strain (data not shown).

The effect of *L. plantarum* supernatant on *P. aeruginosa* and SMG respiratory infections was evaluated after 1, 3, and 5 days of nebulizations. Lung bacterial cell counts after the nebulizations with *L. plantarum* supernatant for 1 or 3 days were not different from controls for strains PaR, PaS, and Paqsc (Figure 7A). *L. plantarum* supernatant significantly reduced lung bacterial cell counts after 5 days of nebulizations in the groups of mice infected with the *P. aeruginosa* strains PaR and PaS, while no differences were noticed for Paqsc. When bacterial loads in the lower respiratory tract of infected mice were compared, significant differences were detected between the strains after 1 or 3 (Figure 7A) days of nebulizations with *L. plantarum* supernatant. The counts of PaR and PaS were higher than Paqsc. In contrast, lung bacterial cell counts in mice treated with *L. plantarum* supernatant for 5 days were not different when PaR, PaS, and Paqsc strains were compared (Figure 7A).

The elastase release in BAL samples was also determined in the infections produced by *P. aeruginosa* strains and SMG (Figure 7B). The release of elastase in mice infected with the three strains in experiments with SMG challenge was slightly higher than the observed when *P. aeruginosa* was used alone: PaR 1.12 vs. 0.83, PaS 0.87 vs. 0.65, and Paqsc 0.76 vs. 0.48 (Figure 7B), indicating an increased virulence of *P. aeruginosa* strains in the presence of SMG. The elastase release in Paqsc-challenged mice was significantly lower than that observed in the groups infected with the PaR or PaS strains, and it was higher in the BAL of animals infected with PaR than in those challenged with *P. aeruginosa* PaS (Figure 7B).

The effect of *L. plantarum* supernatant on elastase release in BAL samples was also determined for the three *P. aeruginosa* strains administered with SMG. The nebulization of mice infected with PaR, PaS, and Paqsc (Figure 7B) with *L. plantarum* supernatant for 1 day did not induce changes in the elastase levels in BAL samples. However, significantly reduced levels of elastase release in BAL were found after 3 and 5 days of *L. plantarum* supernatant treatment, with the effect more remarkable on day 5.

The number of leukocytes in BAL samples to measure the inflammatory response was also evaluated in the infections produced with the three *P. aeruginosa* strains administered with SMG (Table 7). The challenge of mice with *P. aeruginosa* strains significantly increased the number of BAL leukocytes compared to basal levels, the most remarkable effect was observed in animals infected with PaR strain and SMG. As observed with other parameters, the BAL leucocyte counts in mice infected with the PaR and PaS strains in experiments with SMG challenge were higher than the observed when *P. aeruginosa* was used alone: PaR 8.3 × 10^3^ vs. 5.3 × 10^3^ and PaS 5.2 × 10^3^ vs. 4.4 × 10^3^. In contrast, no differences were detected in BAL leucocytes in mice infected with the Paqsc strain with (3.5 × 10^3^) or without (3.3 × 10^3^) SMG.

**Table 7:** Effect of *L. plantarum* supernatant on respiratory infections produced by *P. aeruginosa* strains and SMG.

***P. aeruginosa* strains**	**BAL leukocytes**			
	**No treatment**	***L. plantarum*** **supernatant 1d**	***L. plantarum*** **supernatant 3d**	***L. plantarum*** **supernatant 5d**
PBS	2.5 × 10^1^ ± 0.4	-	-	-
PaR+SMG	8.3 × 10^3^ ± 0.5^†^	2.1 × 10^3^ ± 0.3^*^	1.9 × 10^2^ ± 0.4^*^	3.1 × 10^2^ ± 0.3^*^
PaS+SMG	5.2 × 10^3^ ± 0.6^†^	1.9 × 10^3^ ± 0.3^*^	1.7 × 10^2^ ± 0.2^*^	1.2 × 10^2^ ± 0.2^*^
Paqsc+SMG	3.5 × 10^3^ ± 0.4^†^	1.4 × 10^3^ ± 0.4^*^	1.1 × 10^2^ ± 0.2^*^	1.1 × 10^2^ ± 0.2^*^

Leukocytes counts in BAL samples. Data represent the mean ± SD of triplicated experiments. Asterisks show statistical significance between the indicated groups and non-lactobacilli treated controls in the same row, ^*^p <0.05. Symbols show statistical significance between the indicated groups and controls in the same column, ^†^p <0.05.

The treatment with *L. plantarum* supernatant significantly reduced the levels of BAL leukocytes after 1, 3, and 5 days for the three *P. aeruginosa* strains administered with SMG. The lowest number of leucocytes in mice infected with PaR, PaS, and Paqsc strains and treated with *L. plantarum* supernatant was observed on day 5 (Table 7). The analysis of the different populations of BAL leucocytes showed that for PaR and SMG-infected mice, 5% were neutrophils, while 95% corresponded to macrophages. In animals challenged with PaS and SMG, BAL samples contained 20% neutrophils and 80% macrophages, while in mice infected with Paqsc and SMG, the samples contained 80% neutrophils and 20% macrophages. The treatment with *L. plantarum* supernatant significantly changed the proportion of neutrophils and macrophages in BAL in both PaR and SMG and PaS and SMG-infected mice.

A total of 60% neutrophils and 40% macrophages were found in the first group, and 70% neutrophils and 30% macrophages were found in the latter group. Notably, the treatment with *L. plantarum* supernatant did not induce any changes in the proportion of neutrophils and macrophages in the BAL of mice infected with *P. aeruginosa* Paqsc and SMG.

**Figure 8:**
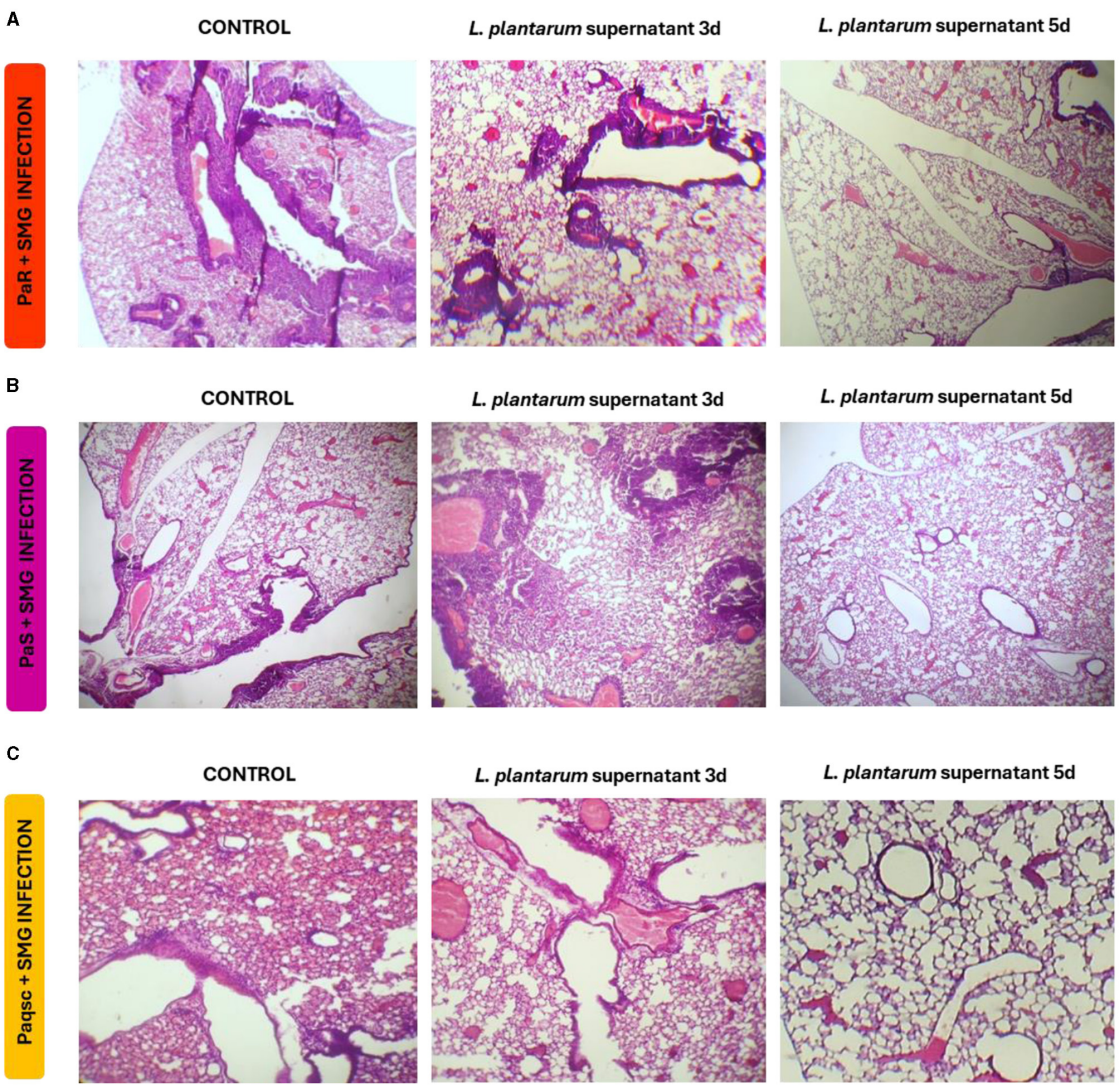
Effect of *L. plantarum* supernatant on respiratory superinfections produced by *P. aeruginosa* strains and SMG. The effects of *L. plantarum* supernatant on respiratory infections produced by different *P. aeruginosa* strains (Paqsc, PaS, and PaR) and SMG are shown. The histology of the lungs was determined. **(A–C)** Lung histopathological analysis at different points post-infection and treatment with *L. plantarum* supernatant for PaR, PaS, and Paqsc plus SMG, respectively. Data were obtained from duplicated experiments (*n* = 3 per experiment).

Lung histology was also studied to evaluate the tissue damage induced by *P. aeruginosa* and SMG infections (Figure 8 and Table 8). The three strains administered with SMG could produce higher lung tissue injury compared to infections with *P. aeruginosa* alone. It was also detected that PaR, PaS, and Paqsc administered with SMG produced different degrees of lung injury severity (Table 8). The PaR strain with SMG-induced hemorrhage, edema, and necrosis was accompanied by intense congestion and inflammatory infiltrates both in the alveolar and interstitial compartments (Figure 8A). Animals challenged with *P. aeruginosa* PaS and SMG also showed edema, congestion, hemorrhage, and inflammatory infiltrate in the alveolar and lung interstitial compartment that were lower than the observed in PaR and SMG-challenged mice (Figure 8B). The histopathological analysis also revealed that the infection produced by the mutant Paqsc strain was much milder when compared to the PaR and SGM infection but similar to the PaS and SMG challenge in some parameters such as edema, hemorrhage, and interstitial inflammatory infiltrate (Figure 8C and Table 8). When the effect of *L. plantarum* supernatant was evaluated, it was determined that after 1 day of treatments, there were no changes in lung tissue damage compared to the respective controls for any of the *P. aeruginosa* strains, as shown in the microscopic observations (Figure 8) and the scores (Table 8). However, the nebulization with *L. plantarum* supernatant for 3 or 5 days significantly decreased lung damage in animals infected with any of the three *P. aeruginosa* strains and SMG. The most notorious effect was found within 5 days of treatment.

**Table 8:** Effect of *L. plantarum* supernatant on respiratory infections produced by *P. aeruginosa* strains and SMG.

**Treatment**	**Edema**	**Necrosis**	**Hemorrhage**	**Congestion**	**Alveolar inflammatory infiltrate**	**Interstitial inflammatory infiltrate**
PaR + SMG	++	+	++	+++	+++	+++
PaR + SMG + *L. plantarum* supernatant 1d	++	+	++	+++	+++	+++
PaR + SMG + *L. plantarum* supernatant 3d	++	0	++	++	++	++
PaR + SMG + *L. plantarum* supernatant 5d	+	0	0	+	+	++
PaS + SMG	++	0	+	++	+	++
PaS + SMG + *L. plantarum* supernatant 1d	++	0	+	++	+	++
PaS + SMG + *L. plantarum* supernatant 3d	++	0	+	+	+	+
PaS + SMG + *L. plantarum* supernatant 5d	+	0	0	+	0	+
Paqsc + SMG	++	0	++	+	+	++
Paqsc + SMG + *L. plantarum* supernatant 1d	++	0	+	+	+	+
Paqsc + SMG + *L. plantarum* supernatant 3d	+	0	+	+	0	+
Paqsc + SMG + *L. plantarum* supernatant 5d	+	0	0	0	0	0

Histopathological score of lung samples. The effects of *L. plantarum* supernatant on respiratory superinfections produced by different *P. aeruginosa* strains (Paqsc, PaS, and PaR) and SMG are shown. Lung histopathological analysis at different points post-infection and treatment with *L. plantarum* supernatant was determined. The degree of edema, necrosis, hemorrhage, congestion, alveolar inflammatory infiltrate, and interstitial inflammatory infiltrate was scored on scales from 0 to +++: absence (0), mild focal (+), moderate to severe focal (++), and severe throughout the lung (+++). Data was obtained from duplicated experiments (*n* = 3 per experiment).

## Discussion

**Figure 9:**
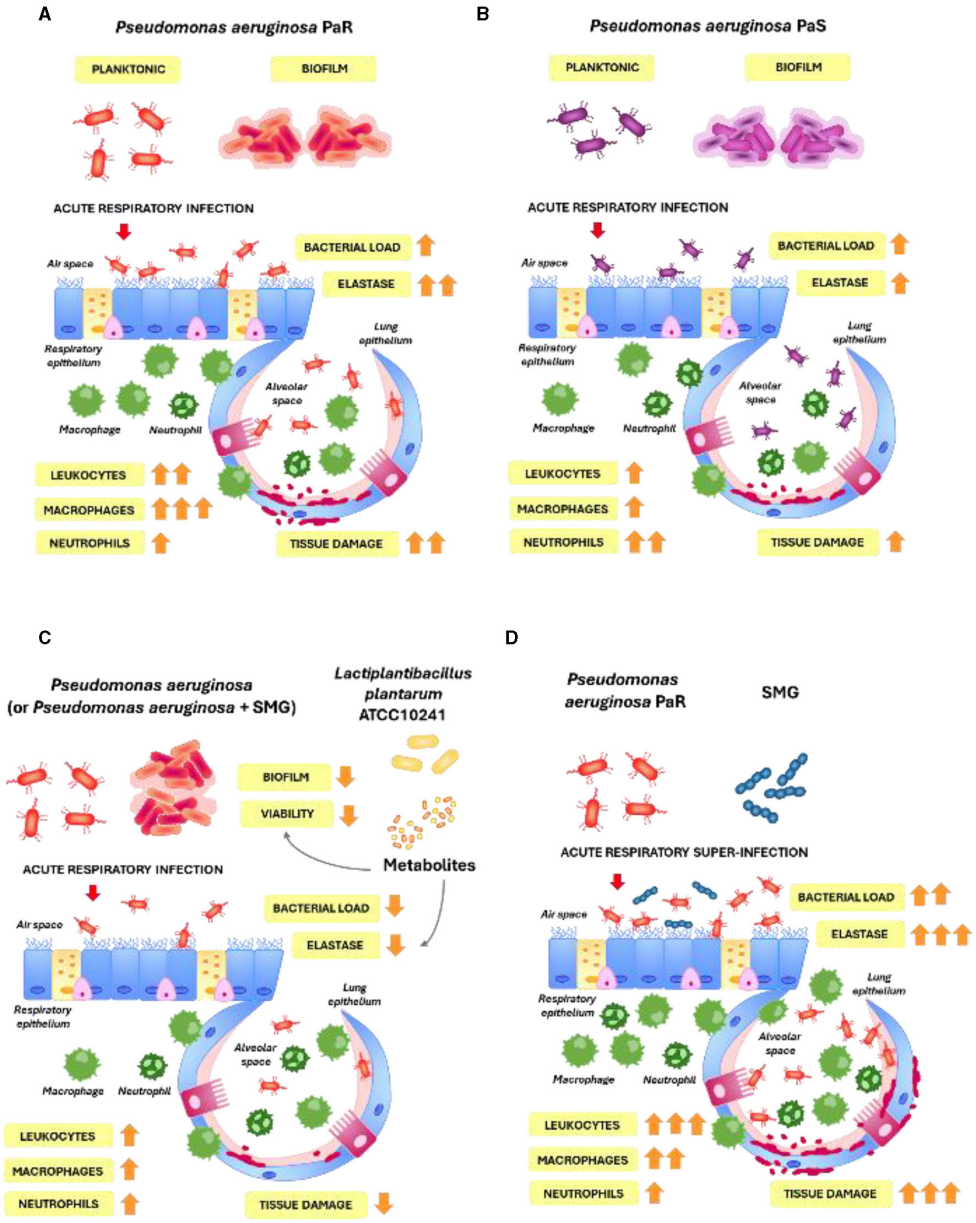
Ability to form biofilms and respiratory infections produced by *P. aeruginosa* PaR **(A)** and PaS **(B)**. Effect of *L. plantarum* supernatant on biofilm formation and respiratory infections produced by *P. aeruginosa* **(C)**. Respiratory superinfections produced by *P. aeruginosa* and SMG **(D)**.

The bacterium *P. aeruginosa* is a ubiquitous microorganism encountered in the environment that usually does not cause problems to human health. However, *P. aeruginosa* is also a formidable opportunistic pathogen that can cause severe infections in susceptible hosts such as those with chronic lung diseases, including CF patients (Faure et al., 2018). Respiratory infections by *P. aeruginosa* in CF patients can be produced by phenotypically distinct strains that vary in the virulence factors and biofilm expressions as well as in their antibiotic resistance (Rossi et al., 2018, 2020). Although it is generally assumed that multi-drug-resistant *P. aeruginosa* strains are less frequently associated with infection, inflammatory responses, and mortality than susceptible strains (Horcajada et al., 2019), it was suggested that some resistance mutations are not associated with fitness/virulence reduction (Skurnik et al., 2013; Pacheco et al., 2017). Furthermore, it was demonstrated that multi-drug-resistant *P. aeruginosa* strains could develop compensatory mutations that allow them to preserve their virulence (Suárez et al., 2010). In line with these studies, we demonstrated here that both PaR and PaS isolated from CF patients, when administered by the nasal route, can produce infections in the respiratory tract of mice, triggering inflammatory responses and inducing lung damage. Notably, the capacity of PaR to induce inflammation and lung damage (Figure 9A) was superior to the observed for PaS (Figure 9B). In addition, the respiratory infection caused by the Paqsc strain was less severe than the one induced by PaR and PaS. These results were expected since it was shown that AHL signaling is an important virulence factor in *P. aeruginosa* (Whiteley et al., 1999; Schuster et al., 2003). There are two AHL quorum-sensing systems in this bacterium, LasI-LasR and RhlI-RhlR. The LasI is responsible for synthesizing 3O-C12-HSL, while RhlI is responsible for synthesizing C4-HSL. The production of these two molecules, which are regulators of gene expression and virulence, is not produced by Paqsc. Then, we conducted experiments using three *P. aeruginosa* strains that produce respiratory infections with a different degree of severity.

It should be considered that the differences in the virulence of *P. aeruginosa* in patients with CF are given by the moment the pathogens are studied. Increased antibiotic resistance and decreased virulence are generally observed during chronicity (Faure et al., 2018). In our experimental model, infections with PaR and PaS occurred acutely, thus it is expected that the microorganisms would not present the transcriptional changes induced in the CF lung during chronicity, allowing them to cause acute infections. In this regard, it was reported that the expressions of some virulence factors, such as proteases and secretion systems, in bacteria growing in biofilms, are maintained at similar levels to those found in free-living cells (Rossi et al., 2018; Kordes et al., 2019), whereas other virulence factor genes experience a reversion of regulation when the environmental conditions of chronic states are eliminated (Bartell et al., 2019). These results indicate that both PaR and PaS are strains with intrinsic virulence capable of inducing both acute infections, as demonstrated in the *in vivo* experiments in this study, and chronic infections, as observed in the patients from which they were isolated (Delgado et al., 2018). It is also possible to conclude that PaR would be intrinsically more virulent than PaS. This statement is supported by our results showing that PaR produced higher levels of elastase in the respiratory tract of infected mice than PaS. *P. aeruginosa* produces several secreted proteases, including the elastase, that allow the bacteria to interact with a wide range of molecules in the host, causing direct tissue damage. Therefore, elastase is considered a key virulence factor in the pathogenesis of acute infections (Tang et al., 1996; Le Berre et al., 2011; Faure et al., 2018).

In addition, we evaluated whether the respiratory administration of the cell-free supernatant of *L. plantarum* ATCC^®^ 10241^TM^ induced some beneficial effect in the infection and inflammatory response of PaR, PaS, and Paqsc *in vivo*, considering our previous data demonstrating its potential to inhibit the multiplication, viability and, AHL synthesis of *P. aeruginosa* strains isolated from wounds (Valdéz et al., 2005). These results were confirmed and extended by *in vitro* studies in this study, which used pathogen strains isolated from the respiratory tract of CF patients. Although clinically controlled trials demonstrated that probiotic supplementation could diminish pulmonary exacerbations and enhance the quality of life of children with CF (reviewed in Neri et al., 2019), the mechanism of micro-microbe and microbe-host interaction in this context has not been explored in depth. Notably, most of the studies investigating the potential of lactobacilli as an alternative antimicrobial therapy against *P. aeruginosa* in CF were performed in *in vitro* studies, while fewer were explored *in vivo*. In addition, most of the studies evaluated orally administered probiotic strains that would exert their beneficial effects on the respiratory tract through the modulation of the gut–lung axis (Neri et al., 2019; Villena and Kitazawa, 2020). However, it seems more rational that the beneficial effect of probiotics might be potentiated by directly administering them into the respiratory tract. In this way, bacteria and/or their metabolites would have the possibility to directly act on the lung pathogens and/or modulate the local immune responses. In this study, we offer solid data to support this statement by demonstrating that the nebulization with *L. plantarum* supernatant can reduce the susceptibility of mice to respiratory infections induced by *P. aeruginosa* strains isolated from CF patients. In our study, the administration of *L. plantarum* supernatant reduced the lung bacterial cell counts, diminished lung tissue damage, and differentially regulated the respiratory immune response in mice infected with PaR or PaS as well as the less virulent strain Paqsc (Figure 9C).

*L. plantarum* supernatant treatment reduced the inflammatory damage and differentially regulated the number and proportions of leucocytes in the respiratory tract after the infection with PaR, PaS, and Paqsc. Notably, the elastase from *P. aeruginosa* has been associated with augmented neutrophilic inflammation and lung damage during acute infection in mice (Qu et al., 2018). It was reported that mice infected with high elastase-producing strains have elevated inflammation and damage in the respiratory tract associated with high levels of MPO, IL-1β, IL-6, and KC (Zupetic et al., 2021). Furthermore, high elastase activity is common in *P. aeruginosa* strains isolated from severely ill patients (Zupetic et al., 2021). In mice infected with PaR, higher levels of elastase and lung damage were observed, but a lower proportion of neutrophils were detected. The smaller amounts of neutrophils in the respiratory tract of PaR-infected mice compared with animals receiving PaS or Paqsc could be associated with higher cellular necrosis induced by the most virulent strain. It was shown that rhamnolipids produced by pathogenic *P. aeruginosa* cause the lysis of several cellular human immune cells, including polymorphonuclear leukocytes (Van Gennip et al., 2009). Furthermore, in a pulmonary model of *P. aeruginosa* infections in mice, it was demonstrated that bacteria with a reduced ability to produce rhamnolipids did not cause the necrotic death of neutrophils and were cleared more efficiently from the lungs of infected mice.

The respiratory administration of the cell-free supernatant of *L. plantarum* ATCC^®^ 10241^TM^ was not only effective in increasing the resistance of mice to the challenge with *P. aeruginosa* but also reduced the lung bacterial loads and the inflammatory damage in mice infected with *P. aeruginosa* and SMG. In an *in vitro* biofilm model, it was shown that the coculture of *P. aeruginosa* DWW2 with *S. anginosus* 3a, both pathogens isolated from CF patients, resulted in significantly higher numbers of streptococci than the observed in monocultures (Waite et al., 2017). In addition, *P. aeruginosa* grown in the presence of streptococci had increased expression of virulence factors (pyocyanin) and caused a higher pathogenicity in *Galleria mellonella*. In line with these previous studies, we showed here that the co-infection of mice with *P. aeruginosa* and SMG significantly augmented the bacterial loads in the lungs and potentiated the inflammatory response, resulting in greater damage of the lung tissue than the observed in animals infected only with *P. aeruginosa* (Figure 9D). The SMG (*S. anginosus*) isolated from CF patients exacerbated the respiratory infections induced by the PaR, PaS, and Paqsc strains. Notably, the *in vitro* studies performed in this study showed an opposite effect when the interaction of PaR, PaS, and Paqcs with SMG was evaluated. SMG inhibited the biofilm formation of *P. aeruginosa* strains, and in *in vitro*, bacteria grow together and cannot be isolated in separate niches for a short period of time. Then, a direct inhibition is easier than when these interactions occur *in vivo*, in which longer interactions are expected, and other bacteria from the local microbiota and host factors are present. Studies of *P. aeruginosa* biofilm formation on CF-derived airway cells showed that *Streptococcus constellatus* induced no effect in the *in vitro* model. However, these streptococci are associated with the onset of pulmonary exacerbations in CF patients (Price et al., 2015). Thus, these data indicate that *in vitro* studies evaluating the interactions of CF pathogens should be interpreted in context since a similar effect will not necessarily be observed *in vivo*, in which a complex network of microbial and host interactions occurs.

Few studies reported the beneficial effects of probiotics on respiratory superinfections. Our previous studies demonstrated that the nasal administration of *Lacticaseibacillus rhamnosus* CRL1505 (Clua et al., 2017, 2020) or *Ligilactobacillus salivarius* FFIG58 (Elean et al., 2023) to infant and adult immunocompetent mice can modulate the local immune response and increase the resistance to a virus-bacteria respiratory superinfection. We demonstrated the benefits of nasally administered probiotics in respiratory superinfection by showing a higher resistance to primary infection with respiratory syncytial virus (RSV) and secondary infection with *Streptococcus pneumoniae*. These beneficial effects were associated with the higher capacity of probiotic-treated animals to limit the growth of pathogens and efficiently regulate TLR3-mediated lung inflammation (Clua et al., 2020; Elean et al., 2023). To the best of our knowledge, the potential beneficial effects of nasally administered probiotics and/or their metabolites to improve the resistance to a bacterial respiratory superinfection have not been reported before. Then, the studies presented here show for the first time the ability of an *L. plantarum* supernatant treatment to favorably impact the *P. aeruginosa* and SMG respiratory superinfection, allowing the reduction of bacterial loads in the lungs, a more controlled inflammation, and lower damage to the lung tissue. Although the nasal administration of probiotic strains, such as *L. rhamnosus* CRL1505 (Clua et al., 2020) or *L. salivarius* FFIG58 (Elean et al., 2023), could have beneficial effects on *P. aeruginosa* and SMG respiratory superinfection, it should be considered that the treatment will be applied to CF patients in which the administration of viable microorganisms could represent a risk. Then, *L. plantarum* supernatant can offer a safer alternative. Another point of contrast with our previous publications is that the CRL1505 and FFIG58 strains were administered preventively before the induction of RSV and *S. pneumoniae* superinfection and were associated with a differential modulation of alveolar macrophages that coordinated an improved innate respiratory immune response against both pathogens (Clua et al., 2020; Elean et al., 2023). Here, *L. plantarum* supernatant was administered after the challenges with the pathogens. The results allow to speculate that the *L. plantarum* supernatant, which contains a complex mixture of compounds with antimicrobial capacities including lactic, butyric, acetic, and succinic acids, H_2_O_2_, benzoic acid, 5-methyl hydantoin, 2,5-mevalonolactone, and isobutyl piperazinedione, would exert antagonistic effects directly on *P. aeruginosa* and SMG, allowing a reduction of their multiplication and biofilm formation and indirectly to a more efficient performance of the local immune system.

The limitations of our study open the doors for future investigations. An interesting question for future research is to determine whether the effect of the supernatant on lung inflammatory damage is due only to its ability to inhibit the growth and biofilm formation of the PaR and PaS strains or whether it can also exert a direct immunomodulatory effect. Kinetic studies of different immune cell populations, cytokines, and chemokines in the respiratory tract could provide valuable information. In addition, it is necessary to test whether the *L. plantarum* supernatant can exert a beneficial effect on chronic *P. aeruginosa* infections in *in vivo* models. These are studies that we intend to explore in the immediate future. Furthermore, clinical studies in CF patients that demonstrate both the efficacy and the security of *L. plantarum* supernatant are necessary.

## Conclusion

Alternative or adjuvant therapies that minimize direct bacterial damage to the host and enhance protective responses can improve infection outcomes in CF patients. Such therapies are particularly needed considering the alarming rise in drug resistance in chronic pulmonary infections. In this study, we provided evidence supporting the potential use of the cell-free supernatant of *L. plantarum* ATCC^®^ 10241^TM^ to induce beneficial effects of respiratory infections and the inflammatory response by *P. aeruginosa*, either alone or in combination with SMG. Although further mechanistic studies are necessary, our results show that the cell-free supernatant of *L. plantarum* ATCC^®^ 10241^TM^ presents a promising adjuvant alternative for treating *P. aeruginosa* respiratory infections and superinfections in CF patients.

## Data Availability

The original contributions presented in the study are included in the article/Supplementary material, further inquiries can be directed to the corresponding authors.
