# Supplementary figures and images for: Effect of Lactiplantibacillus plantarum cell-free culture on bacterial pathogens isolated from cystic fibrosis patients: in vitro and in vivo studies
Source: Front Microbiol. 2024 Sep 16;15:1440090. doi: 10.3389/fmicb.2024.1440090 (PMC11439784; doi:10.3389/fmicb.2024.1440090)

## Slide 1
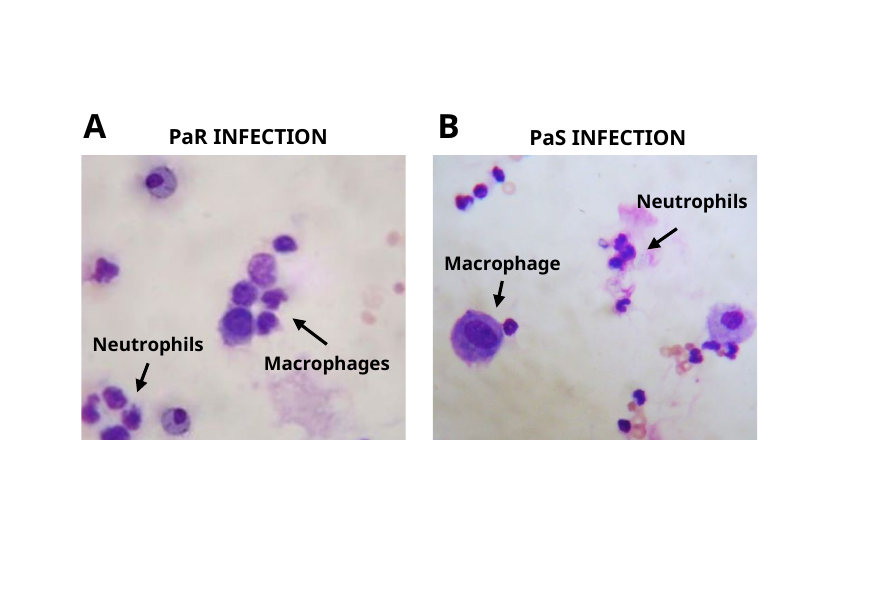

A
B
PaR INFECTION
PaS INFECTION
Neutrophils
Macrophage
Neutrophils
Macrophages

Supplement: Supplementary file 1 [file Presentation_1.pptx]

## Slide 1
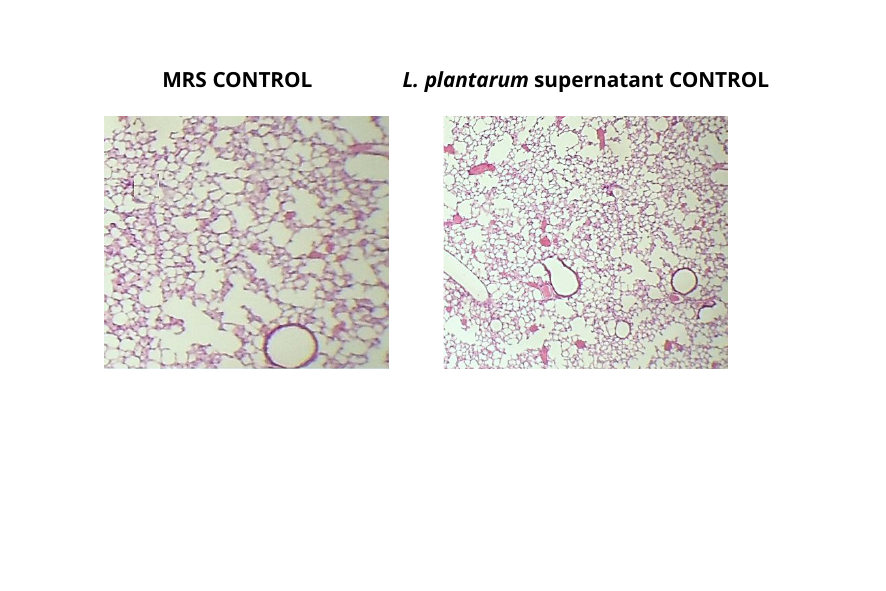

MRS CONTROL
L. plantarum supernatant CONTROL

Supplement: Supplementary file 2 [file Presentation_2.pptx]
